# Laser, Intense Pulsed Light, and Radiofrequency for the Treatment of Burn Scarring: A Systematic Review and Meta-Analysis

**DOI:** 10.3390/ebj4020013

**Published:** 2023-03-23

**Authors:** Yubing Bai, Yiqiu Zhang, Wei Ni, Min Yao

**Affiliations:** 1 Department of Plastic and Reconstructive Surgery, Shanghai Ninth People’s Hospital, Shanghai Jiao Tong University School of Medicine, Shanghai 200011, China; 2 Britton Chance Center for Biomedical Photonics, Wuhan National Laboratory for Optoelectronics, Huazhong University of Science and Technology, Wuhan 430000, China

**Keywords:** burn scarring, laser therapy, IPL, timing and method of photo-electric therapy

## Abstract

Burns and scarring are considered some of the greatest problems in public health because of their frequent occurrence. Today, photo-electric technology shows promising results in the treatment of burn scars. Over the years, more clinical trials and more technologies for scarring have emerged. The aim of this study was to determine better timing and methods of photo-electric therapy for burn scars. This study was registered in PROSPERO (CRD42023397244), following the PRISMA statement, and was carried out in concordance with the PRISMA checklist. In October 2022, we searched PubMed.gov, Embase, and the Cochrane library (1980–present) for published studies related to the photo-electric treatment of burn scars. Two review authors independently selected the studies, extracted the data, assessed the risk of bias among the studies included, and carried out NIH assessments to assess the certainty of the evidence. A third review author arbitrated any disagreements. Our research included 39 studies. We found evidence suggesting that photo-electric therapy between six months and one year offers significantly better outcomes than treatment of scarring after one year. The evidence also suggests the use of IPL for the treatment of early burn scarring. However, it is important to emphasize that the scientific evidence remains insufficient. We need more clinical trials of higher quality and with less heterogeneity to confirm our results.

## 1. Introduction

Burn scars are considered one of the greatest problems in public health [1,2]. Hypertrophic scarring occurs in 30 to 90 percent of patients following burns [3,4,5]. Most burn patients have to suffer physical pain and pruritus in the first stage. As long-term effects, dysfunction and aesthetic deformations in some severe cases also negatively impact patients’ self-confidence, making them feel inferior. Today, first-line therapy for scarring includes surgery, pression therapy, silicone sheets and gel formulations, intralesional pharmacologic treatments, and many others [6,7,8]. However, the recurrence rate of scars after surgery is high at up to 45–100% [9,10,11]. Intralesional pharmacologic treatments are also commonly used, including triamcinolone acetonide (TAC) and fluorouracil (FU) [12,13]. Overall, burn scar characteristics can certainly be improved [14,15,16,17] but also cause many side-effects. For example, TAC can cause atrophy, hypopigmentation, hypertension, hirsutism, and even Cushing’s syndrome [18], and FU may also have myelosuppression activities, causing leukopenia, infection, anemia, and other side effects. Additionally, some treatments cannot obtain satisfactory results. Other methods, including radiotherapy, cryotherapy, and massage therapy, are not commonly used clinically for a variety of reasons [9,19,20]. Meanwhile, photo-electric technology, which shows promising results in the treatment of burn scars [21], produces photophysical (such as thermal, mechanical, and electromagnetic) and photobiological effects (such as photochemistry and photobiological regulation) by skin exposure.

Today, the increasing interest in photo-electric therapy is making a great difference in the treatment of scarring. Photo-electric therapy in scarring includes pulsed dye lasers (PDLs), neodymium-doped yttrium aluminum garnet (Nd: YAG) lasers, low-level lasers (LLLT), intense pulsed light (IPL), ablative fractional carbon dioxide lasers (CO_2_AFL), and radiofrequency (RF). On Pubmed.gov, over two thousand results since 1967 can be found for the photo-electric treatment of scars. However, nearly 1800 of these results were published after the year 2000, and over a thousand were published in the last decade. Thus, in recent years, more clinical trials have been conducted and more technologies for scarring have emerged. These technologies include narrow-spectrum intense pulsed light (DPL) and Q-switched frequency-doubled Nd: YAG lasers. Due to the abovementioned factors, photo-electric treatment has become an efficient modality of therapy for burn scars with few side effects. Some systematic reviews have noted that CO_2_AFL is a safe, cost-effective, and efficacious procedure for burn scars [22] that offers objective improvements specifically for chronic burn scars [23]. However, systematic reviews of other treatments are still scarce. We located a systematic review about the effectiveness of laser therapy for hypertrophic burn scars; this review noted that the evidence is not adequate to reach a conclusion [24]. Additionally, we found a systematic review and meta-analysis about surgical scars that showed that laser therapy may be a useful modality to minimize surgical scars when applied earlier on [25]. However, this study only used four articles to perform the meta-analysis, so more research should be carried out to support this result. We also found a systematic review of early laser intervention in scarring [26]. The results were uncertain as to whether early laser treatment can reduce scar formation, and more high-quality research is needed for a definitive conclusion. There were also some reviews on this topic [27,28]. Globally, there are still many deficits in photo-electric therapy, and no detailed protocol is available. We still have no agreed-upon methods or parameters for the treatment of burn scars, which may cause many side-effects. This study focuses on photo-electric therapy, which can be used in the first period of scarring to prevent progression in a worse direction. We also explore when and how to use these treatments to achieve the most effective outcomes.

## 2. Materials and Methods

### 2.1. Search Strategy

Firstly, this study was registered in PROSPERO (CRD42023397244), following the PRISMA statement, and was carried out in concordance with the PRISMA checklist, which is included in the Supplementary Materials (File S1).

We employed the following search strategy to identify the clinical evidence reported in the biomedical literature: In October 2022, we searched PubMed.gov, Embase, and the Cochrane library (1980–October 2022) for published case reports, clinical studies, clinical trials, controlled clinical trials, and randomized controlled trials related to the photo-electric treatment of burns. We included no restriction for language. The mesh terms we utilized were ‘burn’ AND (‘laser’ OR ‘light’ OR ‘radiofrequency’) AND ‘therapy*’ AND ‘cicatrix’. The details of our search strategy are provided in Table 1.

### 2.2. Selection Inclusion

To be included in the analysis, an original article had to meet the following inclusion criteria: (1) subject: patients who had clinically obvious scars, with more than 50% of the sample featuring scarring due to burns; (2) intervention: treatment of scars needed to involve photo-electric therapy; (3) outcome: Vancouver Scar Scale (VSS) score, Patient and Observer Scar Assessment Scale (POSAS), Visual Analogue Score (VAS), and scar thickness (mm) measured with ultrasonography; (4) control: pretreatment in individuals or other treatments or an untreated area control; (5) study design: randomized controlled trial (RCT), non-randomized control trial, pre–post study of the same person, cohort study, case–control study, and/or comparative study; (6) a mention of scar duration.

**Figure 1:**
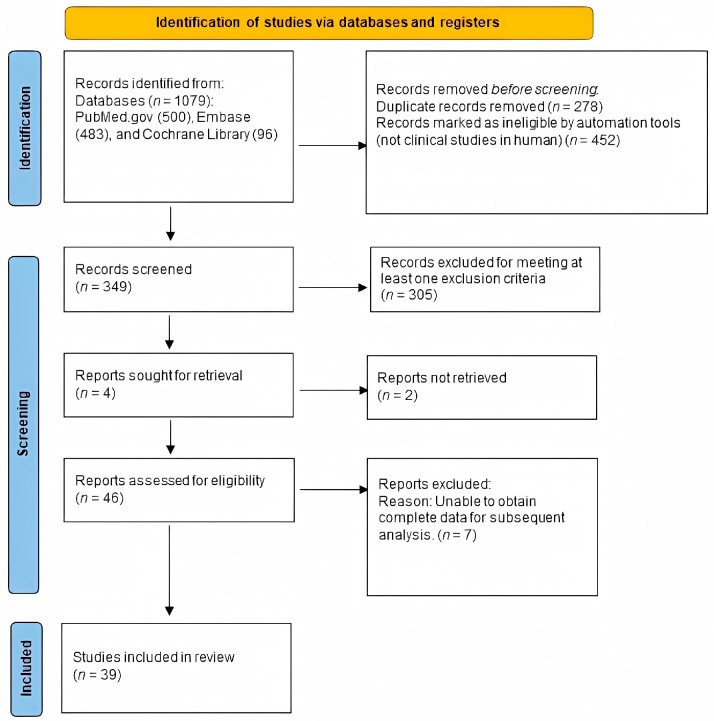
Eligibility of studies for inclusion in the meta-analysis.

The exclusion criteria were as follows: (1) subject: more than 50% of the sample due to etiologies other than burns; (2) intervention: treatment of scars did not involve photo-electric therapy; (3) outcomes: measurement methods did not include the Vancouver Scar Scale (VSS), the Patient and Observer Scar Assessment Scale (POSAS), the Visual Analogue Score (VAS), or scar thickness (mm) measured with ultrasonography; (4) control: no control; (5) study design: case report or case series; (6) no mention of scar duration (Figure 1).

### 2.3. Data Extraction

Two independent investigators browsed all included studies and recorded the features and outcomes of the trials using a data extraction form. The following variables were summarized in a standard Excel file: first author’s name, year of publication, study design, control, duration of follow-up, sample size, country, patients’ baseline characteristics, the type of treatment, the parameters used, the requirements, whether or not any other scar treatments were used concurrently, and the main outcomes (VSS, total POSAS, POSAS-patient, POSAS-observer, VAS, and thickness). If the study used multiple evaluation data, then our selection order was as follows: (1) total POSAS, (2) POSAS-observer, (3) POSAS-patient, (4) VSS, and (5) VAS and thickness. These instruments are the most widely used and objective assessment criteria for burn scars. We also contacted the corresponding authors for more detailed information when the necessary data were not presented in the original study. Discrepancies between investigators were resolved by discussion and consensus.

### 2.4. Methodological Quality Assessment of Included Studies

The quality assessment for all studies was performed using the study quality assessment of the National Heart, Lung, and Blood Institute (NIH) [29]. This assessment has different scales for each type of study, with ratings of good, fair, and poor.

### 2.5. Statistical Analysis

Consensus in China indicates that the length of immature scarring varies greatly between individuals and is dependent on a number of factors. Most scars reach maturity in 6–12 months, but the average immature period for hyperplastic scars can be 22–46 months [30]. As a result, to determine the best time to start treatment and the best method for scar treatment within one year, we analyzed relevant data by dividing the samples into the following groups: scarring for less than six months, scarring for six months to one year, and scarring for longer than one year. We used Review Manager 5.4 to calculate the std. mean difference (SMD) or weighted mean difference (WMD) with a 95% confidence interval (95% CI) for continuous outcomes. As substantial heterogeneity was identified, we used only the random-effects model. A *p*-value less than 0.05 was judged to be statistically significant.

## 3. Results

### 3.1. Identification of Eligible Studies

A flowchart of the literature search process is presented in Figure 1. Our search yielded 349 unique articles. Of these, 46 articles met our inclusion criteria (Figure 1), and 39 articles were available. We found that 12 were cohort studies [31,32,33,34,35,36,37,38,39,40,41,42], 3 were case–control studies [43,44,45], 5 were RCTs [46,47,48,49,50], 4 were non-randomized controlled trials [51,52,53,54], and 15 were in-patient controlled studies [55,56,57,58,59,60,61,62,63,64,65,66,67,68,69]. Table 2 summarizes the characteristics of the 39 studies. These studies were published between 2004 and 2020. The population involved mainly burn scars. The studies were mainly of a good or fair level when assessed by the NIH, suggesting that these studies were of moderate or high quality. Ultimately, we included 22 studies with a total of 916 patients who suffered scarring for over one year, 10 studies with 355 patients who suffered scarring for over 6 months but less than 1 year, and 13 studies with 1101 patients who had scars for up to 6 months.

### 3.2. Time of Intervention

**Figure 2:**
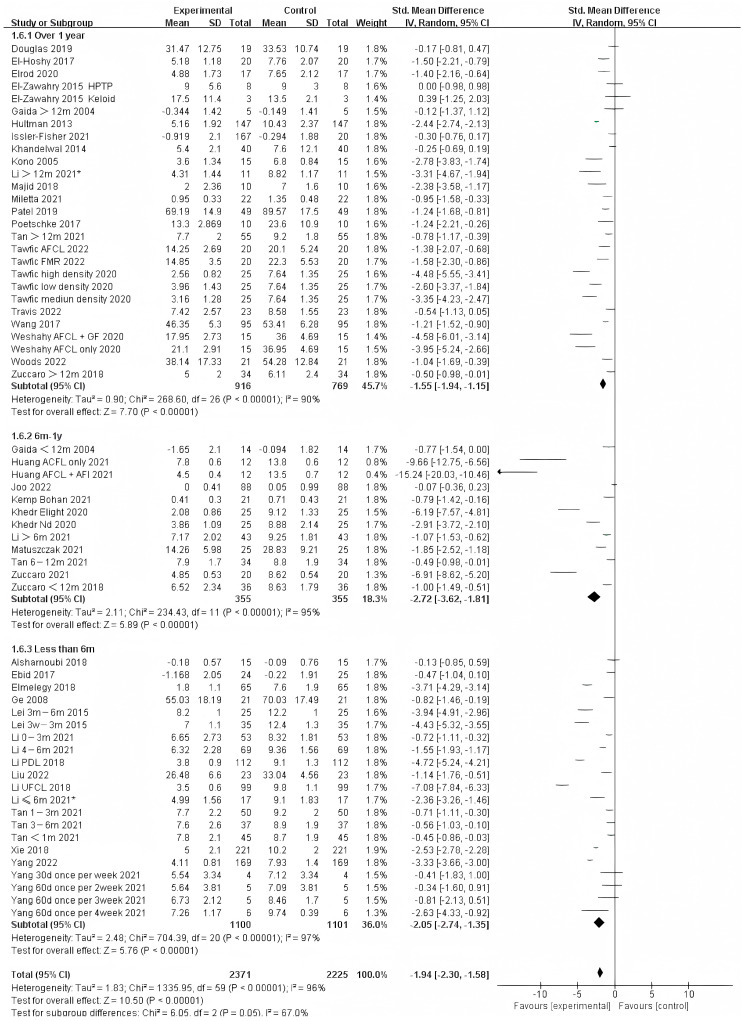
Forest plot showing the effects of photo-electric therapy for burn scars with different scar durations according to VSS, POSAS, thickness, and VAS [31,32,33,34,35,36,37,38,39,40,41,42,43,44,45,46,47,48,49,50,51,52,53,54,55,56,57,58,59,60,61,62,63,64,65,66,67,68,69]. Each trial is represented by a green point, and the size of the point is proportional to the information in that trial. The ends of the horizontal bars denote 95% confidence intervals (Cis). Black diamonds indicate the overall results of all trials. * In order to distinguish between the two Li’s articles published in 2021, we have marked this one with an asterisk [43].

**Figure 3:**
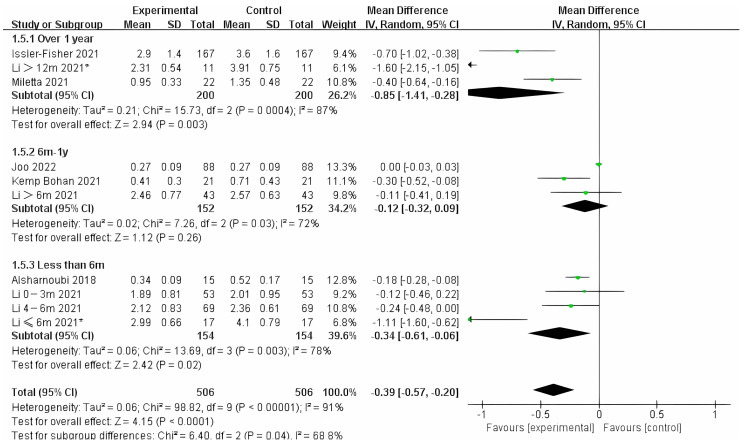
Forest plot showing the effects of photo-electric therapy on the thickness of burn scars with different scar durations [35,43,45,48,53,58,63]. Each trial is represented by a green point, and the size of the point is proportional to the information in that trial. The ends of the horizontal bars denote 95% confidence intervals (Cis). Black diamonds give the overall results of all trials. * In order to distinguish between the two Li’s articles published in 2021, we have marked this one with an asterisk [43].

**Figure 4:**
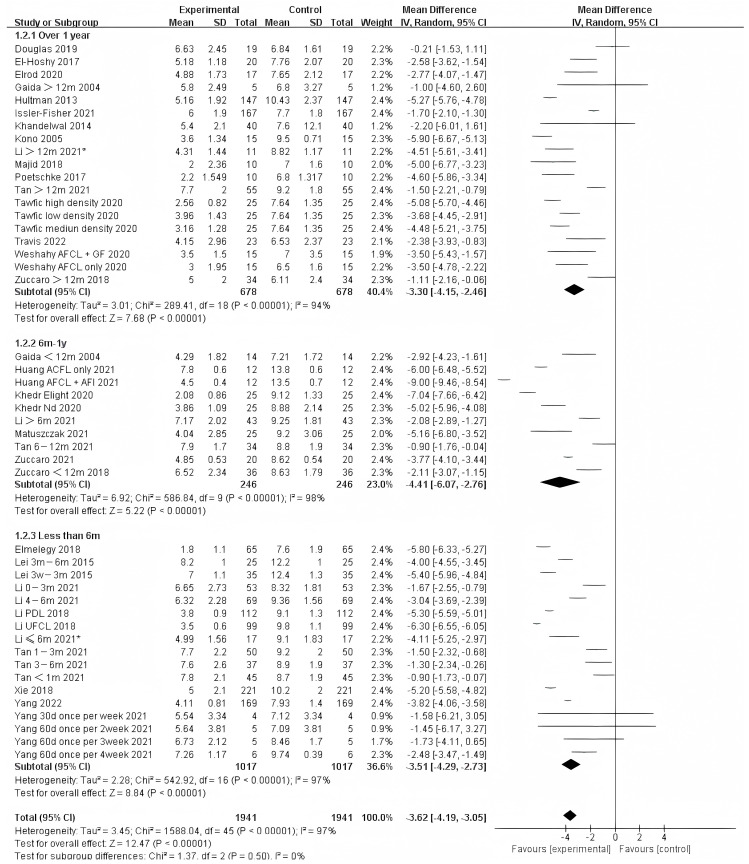
Forest plot showing the effects of photo-electric therapy using VSS for burn scars with different scar durations [31,33,34,35,36,38,39,41,42,43,45,46,50,52,54,55,56,57,59,60,61,62,64,65,67,68,69]. Each trial is represented by a green point, and the size of the point is proportional to the information in that trial. The ends of the horizontal bars denote 95% confidence intervals (Cis). Black diamonds indicate the overall results of all trials. * In order to distinguish between the two Li’s articles published in 2021, we have marked this one with an asterisk [43].

Photo-electric therapy offered significant improvement for each period of burn scarring (Figure 2) (Chi² = 6.05, df = 2 (*p* = 0.05), I² = 67.0%). Furthermore, for the group with scarring for over one year and the group with scarring between six months and one year, there was a significant difference in improvement (Chi² = 5.43, df = 1 (*p* = 0.02), I² = 81.6%) (Figure S1). However, there was no significant difference between the group with less than 6 months of scarring compared to the other two groups. For deeper insight, we also analyzed the improvement using only thickness and VSS. Interestingly, we found that in terms of thickness, the photo-electric therapy presented a significant difference in improvement of the scar over one year (Figure 3) (Chi² = 6.40, df = 2 (*p* = 0.04), I² = 68.8%). However, in VSS, although there was no significant difference between the two groups, scarring less than one year presented a higher effect size than scarring over one year (Figure 4) (Chi² = 1.37, df = 2 (*p* = 0.50), I² = 0%).

### 3.3. Method for Burn Scarring

**Figure 5:**
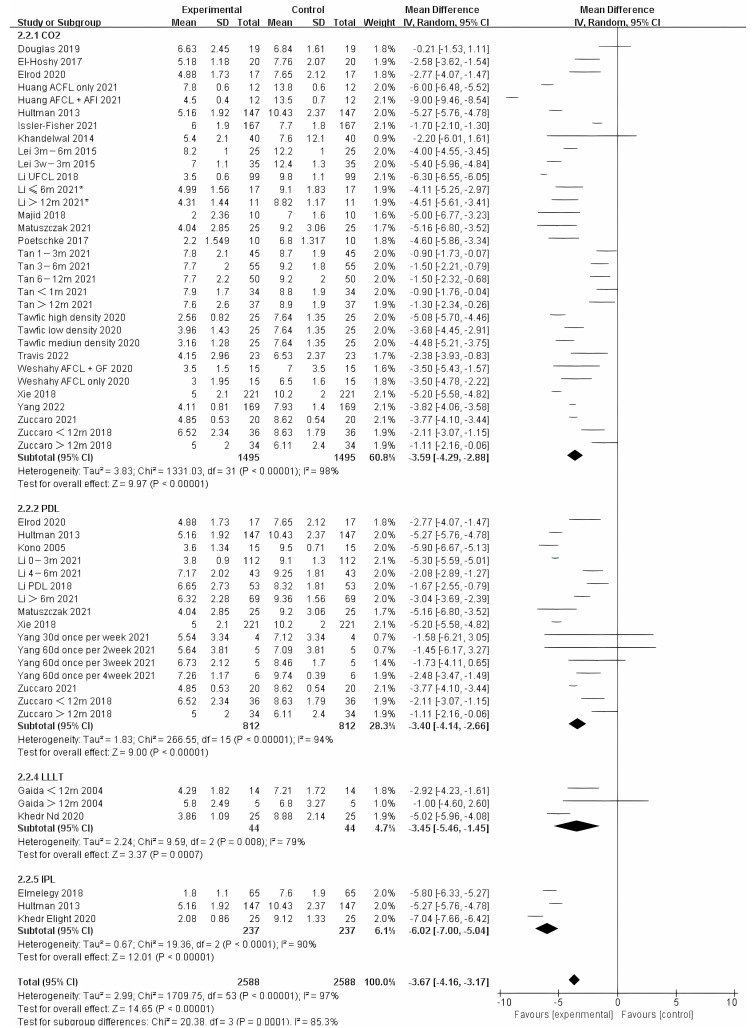
Forest plot showing the effects of different photo-electric therapies for burn scars, using VSS [31,33,34,35,36,38,39,41,42,43,45,46,50,52,54,55,56,57,59,60,61,62,64,65,67,68,69]. Each trial is represented by a green point, and the size of the point is proportional to the information in that trial. The ends of the horizontal bars denote 95% confidence intervals (Cis). Black diamonds give the overall results of all trials. * In order to distinguish between the two Li’s articles published in 2021, we have marked this one with an asterisk [43].

We also analyzed the effects of different photo-electric therapies for all periods of burn scars. The results showed significant differences between the methods (Figure 5) (Chi² = 20.38, df = 3 (*p* = 0.0001), I² = 85.3%). Overall, therapies that included IPL were found to work best.

**Figure 6:**
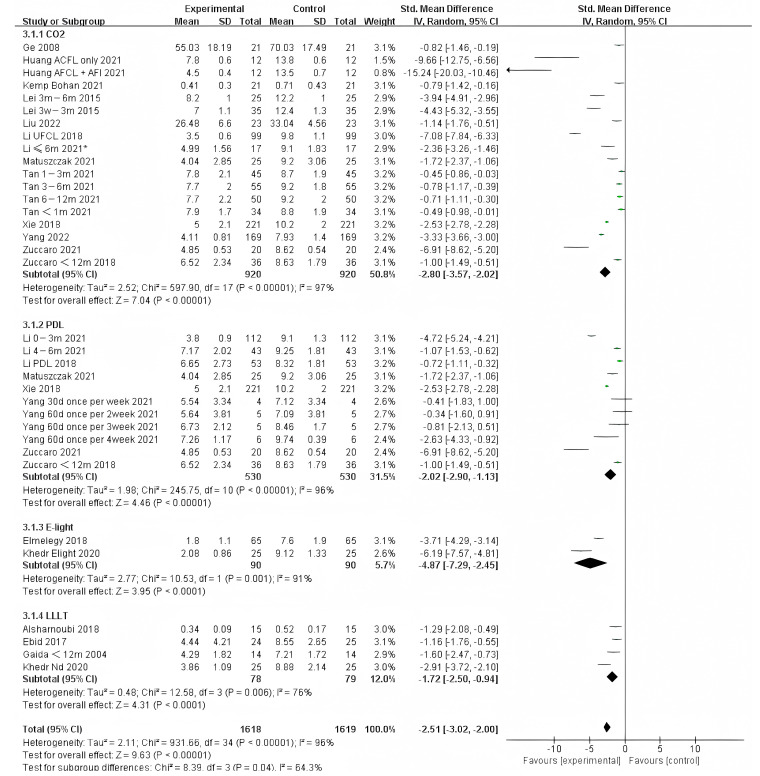
Forest plot showing the effects of different photo-electric therapies for burn scars within 1 year. Each trial is represented by a green point, and the size of the point is proportional to the information in that trial. The ends of the horizontal bars denote 95% confidence intervals (CIs). Black diamonds give the overall results of all trials. * In order to distinguish between the two Li’s articles published in 2021, we have marked this one with an asterisk [43].

For scarring less than one year, E-light (combined radiofrequency and IPL) offered significantly different improvement compared to other therapies. However, IPL was represented in only two studies of the 90 samples. Therefore, more studies on E-light should be performed (Figure 6). Although there was no significant difference between other therapies, it appears that CO_2_ treatment was more effective than PDL.

### 3.4. Publication Bias

Our assessment showed no evidence of significant publication bias based on formal statistical tests (Egger’s test, *p* = 0.056 > 0.05).

## 4. Discussion

In this study, we found that treatments for scarring over six months and one year have significant improvement differences in general presentation compared to other periods of scarring. The formation of scarring can be divided into three stages: inflammation, proliferation, and remodeling. During the first few days after an injury, corresponding to the inflammation stage, a variety of chemokines and vessel active mediators are produced at the site of the injury [70]. Then, in the proliferation stage, vessel active mediators, such as vascular endothelial growth factor (VEGF), from the previous stage induce microvascular scar tissue, which leads to scar proliferation [71,72,73]. The degree of microvascular density and scar hyperplasia are positively correlated [74]. Therefore, theoretically, intervention in this period can reduce angiogenesis, which can help relieve pruritus, contracture scars, and prevent scar growth and dysfunction. Histological analysis showed that the density of blood vessels in scar tissue increases significantly starting at one month after wound healing [74]. Then, hypertrophic scars generally develop in 2~6 months [27]. However, the results vary greatly between individuals due to different factors. Notably, burn scarring, the time of healing, and remodeling can be prolonged [30], so the best time for intervention in burn scarring is within one year, as shown by our results; interventions may also need to be personalized. Poetschke, J et al. [28] published a similar review on the treatment of immature scarring and concluded that a treatment algorithm should be formulated according to each patient’s needs. In conflict with our results, Brewin, M. P et al. [27] proposed that the treatment of PDL should begin before six months, when the scarring remains immature. This difference depends on the definition of immature scarring. As we mentioned, the duration of immature scarring varies greatly between individuals, making it difficult to clearly determine the ideal intervention time. Thus, the best way to deliver treatment is to follow-up with the patient as early as possible and avoid starting treatment too late. Treatment within one year is a good choice based on our results. Lastly, in the remodeling phase, the scar no longer presents redness, and for a hypertrophic or keloid scar, the scar may continue to thicken. This agrees with our outcome that in terms of thickness, photo-electric therapy corresponds to significant differences in improvement of the scar over one year.

In our study, treatment with the addition of IPL offered better improvements than other devices, especially for burn scars treated within one year. IPL therapy is non-invasive, non-surgical, and preliminarily filtered, forming an intense light with a wavelength of 400 to 1200 nm. IPL is not a laser but has similar characteristics to a laser [72]. Through the function of selective photothermolysis, light energy is absorbed by chromophore oxyhemoglobin, which is abundant in the blood vessels, causing photocoagulation of the vascular endothelium. This chromophore can also be absorbed by melanin in the epidermis. Thus, after the application of IPL, melanosomes in the epidermal melasma quickly move to the surface of the skin, undergo desquamation, and take the form of tiny crusts. Li. N et al. [75] used IPL to treat 35 Chinese patients who had a history of skin burns within the past year. The results showed that IPL is effective and safe in Chinese patients with postburn hyperpigmentation and telangiectasia. Meanwhile, as the maximum absorption by collagen occurs in the visible and near-infrared spectra [76], the light can also be absorbed by collagen. It was further confirmed that, with IPL, the activity of fibroblasts is increased, causing upregulation of type-I and type-III collagens at the mRNA and protein levels and rearranging elastin fibers both in vitro and in vivo [77,78]. However, we did not find a convincing systematic review that evaluated the effectiveness of IPL. In the systematic review of Vrijman, C et al. [76], the authors did not find any evidence for the efficacy of IPL therapy, as no study met the inclusion criteria. Zuccaro, Jv [24] found only one study about IPL, which reported mild-to-significant improvement in scarring.

With the development of technology, filtering narrow-spectrum intense pulsed light (DPL) of 500–600 nm through the spectrum at both ends can make treatment energy more concentrated; when the spot is large and uniform, the energy is lower and can more effectively protect normal skin tissue around the scar. This method offers the precision of a laser and the safety of strong pulsed light, greatly improving the curative effects. DPL still contains the absorption peak of hemoglobin, reduces the absorption of light by other tissue, and can use higher energy to block blood vessels; DPL can also inhibit angiogenesis, is more specific than IPL, and leads to less pain than PDL [72,79]. Zhang et al. [79] used DPL to treat 90 patients with scars after 3 weeks but within 1 year. After treatment for 3 months, the pruritus of scars was obviously alleviated. The degree of microvascular regeneration was related to the formation of erythema in the scar, which, in turn, became taller and harder, as well as the level of the hypertrophy of the scar [80]. As a result, we suggest that once the scar heals and appears red, photo-electric therapy that targets neovascularization should be started.

At the same time, the effectiveness of other methods cannot be ignored. Our study still showed a great effect of CO_2_AFL, PDL, LLLT, and RF on scar appearance. The CO_2_AFL can create 3D microthermal damage zones, thereby decreasing wound repair time and adverse reactions. Additionally, the use of fractional carbon dioxide laser treatment for hypertrophic scars can promote a decrease in type I collagen in scar tissue and an increase in type III collagen, which is closer to the collagen structure of normal skin tissue [81]. This treatment can also cause damage to blood vessels, producing scar ischemia and releasing collagenase to break down collagen, while the thermal effect of lasers can also stimulate collagen synthesis and remodeling, which helps to promote collagen remodeling as well as improve the appearance of scars [50]. A recent study compared the effects of starting CO_2_AFL treatment at multiple times after injury. One month after the last treatment, the results showed that CO_2_AFL was more effective for scars after more than 12 months in terms of height and pliability. However, for hardness and redness, scars at 1–3 months presented better results than other groups. The authors suggested that the ideal time point for the initiation of early fractional laser treatment could be within 1 month after injury [38]. However, there are some common side effects of CO_2_AFL, including erythema, seepage, bleeding, swelling, pigmentation, and deterioration of scarring [38,82]. Lower density with moderate laser energy for treating scars was proposed to avoid such problems.

PDL (pulsed dye laser) is the most widely used and effective type of laser for preventing early scarring [83]. According to the principle of selective photothermolysis, hemoglobin has two absorption peaks at 542 and 578 nm. Therefore, a laser at 585 nm will have a noticeable effect on eliminating blood vessels. Meanwhile, through the function of photothermolysis, collagen fibers are heated, and the disulfide bonds are broken, enabling them to be catabolized. In this way, collagen over-deposition can be prevented, stimulating collagen remodeling and allowing for the structure of scar epithelial tissue to be reconstructed [46,84]. However, the efficacy of PDL is limited by the thickness of the lesion. PDL penetrates to a depth of approximately 1.2 mm [85]. The most common side effect of PDL is postdelivery purpura, which persist for up to 7–10 days [41]. When the PDL energy is too high, pigment loss can easily occur [27]. Thus, to treat deeper lesions and reduce the side effects at the same time, more methods for combining other treatments with PDL need to be developed. Recently, Naoaki Rikihisa et al. [86] found that intravenous preadministration of carbonyl hemoglobin vesicles (CO-HbVs) followed by the application of vascular selective laser irradiation to the blood vessels of rabbit pinna reduced thermal damage to the perivascular tissue and partially enhanced vascular damage. In combination with Nd:YAG, PDL first changes hemoglobin into methemoglobin, which can absorb more energy from the Nd:YAG laser and thus penetrate more deeply [84].

LLLT treats scars in a different way by acting on the skin through photobiomodulation (PBM), which is an efficient and safe therapeutic modality for postburn scars. LLLT was found to suppress the viability of fibroblasts, inhibit the proliferation and formation of collagen in skin, and increase apoptosis of fibroblasts through mitochondria [87,88]. LLLT can also improve macrophage migration and phagocytosis independently of TGF-β signaling [89]. At present, LLLT is known to cause no side effects, which is a great advantage in early scar treatment [48]. It is known with certainty that LLLT promotes beneficial effects in the early stages of burn injury. However, we still need more basic and clinical studies to understand the relevant mechanisms and direct the best parameters for each type of burn, each type of skin, and each stage of scarring.

Finally, the mechanism underlying the RF stimulation of collagen fiber remodeling is likely protein denaturation caused by the effects of heat followed by the stimulation of collagen synthesis due to the increased expression of heat shock proteins [90]. With the development of fractional technology, in 2010, fractional microplasma radiofrequency technology (FMRT) was developed and initially used for the treatment of facial scars and rhytids [91]. Pinheiro et al. [92] compared the histological examination of postburn hypertrophic scar tissue treated with RF. The results showed that the treated area featured collagen fiber density in the papillary and reticular dermis similar to that of normal skin. This density was significantly greater in the area with no RF treatment [93]. However, the thermal effects of radiofrequency occur in the deeper layers of the skin, so short-term irritation, edema, and even burns can occur [94]. Additionally, numbness and sensory dullness can occur when thermal coagulation leads to demyelination of sensory nerves [95], so more experienced operators are needed for treatment.

## 5. Conclusions

In conclusion, this study indicated that treatment starting between six months and one year after injury had better outcomes in terms of the general presentation of scarring. Meanwhile, using IPL for burn scarring treatment seems to have better effects than other methods, especially for scarring within one year. We suggest using IPL and, especially, DPL for the treatment of early burn scarring. Notably, the scientific evidence in this area remains insufficient. We need more clinical trials of higher quality and less heterogeneity to confirm our results.

## 6. Limitations

There are still some limitations to this study. First, the majority of studies we included used pretreatment controls. Most burn scars improve over time, but concurrent control experiments in this area are extremely scarce. There is also a lack of higher-quality clinical trials, such as RCTs, and an inability to apply a double-blinded method for laser therapy. Meanwhile, some studies featured very short follow-ups. Significant heterogeneity was also observed among the studies included in this systematic review, including in the parameters, the application of different treatments, and the lack of general assessments evaluating burn scars. As a result, more well-performed, larger RCTs need to be carried out. The various evaluation criteria (POSAS for the patient and observer, VSS, etc.) also need to be standardized to further verify our results.

## Figures and Tables

**Table 1:** Search details on PubMed.gov.

	Participants	Intervention	Study
Mesh word	burn* AND Cicatrix	(laser* OR light OR radiofrequency) AND therap*	Clinical Study in Humans
Free word	(postburn OR ‘post burn’) AND (Scar OR Scars OR Cicatrization OR Scarring OR ‘hypertrophic scar*’ OR keloid)	(‘fractional carbon dioxide’ OR ‘fractional CO_2_′ OR ‘pulsed dye laser’ OR PDL OR Biostimulation OR Photobiomodulation OR ‘low level laser’ OR LLLT OR ‘Neodymium Doped Yttrium Aluminum Garnet’ OR er yag OR ‘erbium‘ OR ‘nd yag’ OR photothermol* OR ‘intense pulsed light’ OR IPL OR radio-frequency) AND (treatment* OR therapeutic*)	

* Vocabulary with this root word.

**Table 2:** Baseline characteristics of patients in the trials included in the meta-analysis.

Author	Year	Study	Control	Follow-Up (Month) after Final Treatment	Sample Size	Gender	Age	Treatment	Other Treatments Combined	Parameter	Device	Outcomes Measure	NIH
		Design				(M/F)	(Years)						
Alsharnoubi [48]	2018	RCT	UAC	3	15	5/10	4.73 ± 1.79	LLLT	NA	Λ = 632.8 ED = 16	Bbravo terza serie HENE laser (ASA s.r.i)	VSS, ST	GOOD
Douglas [50]	2019	RCT	UAC	1.5	19	15/4	29	UFCL	NA	5% density, 300 Hz, 50 mJ	Ultrapulse, Lumenis	VSS, P-POSAS	GOOD
Ebid [49]	2017	RCT	Placebo control	3	49	30/19	31.53 ± 10.14	Pulsed Nd:YAG laser	NA	Λ = 1064, ED = 0.510–1.78, 10–40 Hz, PD = 0.12–0.15, S = 4.47	HIRO3 machine, ASA Laser company, Italy	VAS	GOOD
El-Hoshy [55]	2017	IPCS	PC	2	20	4/16	26.35 ± 9.85	AFCL	NA	stacking, 3; PD = 0.6; spacing, 200 µm	SmartXide DOT^®^; DEKA, Florence, Italy	VSS, POSAS	GOOD
Elrod [31]	2020	cohort (retrospective)	PC	After the final laser session	17	8/9	11.37 ± 4.82	Combined AFCL + PDL (94%), with AFCL only (6%).	NA	PDL firstly: λ = 595, PD = 6–8, PD = 0.45, S = 10, no more than 30% overlap;	PDL: V-beam2; Candela, Wayland, MA, USA; AFCL: DeepFX hand-piece (Ultrapulse, Lumenis)	VSS, POSAS	GOOD
										Secondly AFCL: 2–3% density, 250 Hz, 70–20 mJ per micropulse			
El-Zawahry [51]	2015	NRCT	UAC	3	15	2/13	30.8 ± 11.3	AFCL	NA	30 W, 800 μm spacing, 800 us dwelling time (the first session followed by 30 W, 300 μm spacing, 800 us dwelling time)	NA	VSS, P-POSAS, O-POSAS	FAIR
Elmelegy [56]	2018	IPCS	PC	1	65	49/16	49/16	E-light	NA	IPL: λ = 530/560/580/630/755 (differs from patients’ skin color). ED = 6.25–6.47; S = 8–32. PD = 2–7, pulse delays 15–30 ms. RF: fluence = 10–12 J	Beijing Oriental Wison Mechanical & Electronic Co., Ltd.	VSS	GOOD
Gaida [52]	2004	NRCT	UAC	2	19	14/5	38 ± 13.97 (18–77)	LLLT	NA	λ = 670, ED = 4	Helbo^®^; Gallspach, Austria	VSS	FAIR
Ge [32]	2022	Cohort (retrospective)	PC	3	21	8/13	31.4 (15–47)	UFCL	NA	3–5% density, 30–300 Hz, 20–175 mJ, 1–60 W	Lumenis Ltd., Yokneam, Israel	Total POSAS	GOOD
Huang [57]	2021	IPCS	PC	6	12	7/5	32 ± 11	AFCL	Autologous fat injection in AFI group	8% density, 40~50 mJ/cm^2^, 10–50 W	KL type fractional CO_2_ laser	mVSS	GOOD
Hultman [33]	2013	Cohort	PC	4.65	147	NA	26.9	PDL, UFCL, IPL, Alexandrite laser	NA	PDL: λ = 595, ED = 8.3 ± 1.1 (5.0–10.0), PD = 1.5, S = 7; end point: ecchymosis; AFCL: 15% density, 600 Hz, 15 mJ/micropulse for deep penetration; 150 Hz and 70–90 mJ/micropulse for more superficial ablation;	PDL:Candela V-beam, Wayland, MA, USA AFCL: Lumenis UltraPulse, ActiveFX, DeepFX handpieces, Santa Clara, CA, USA IPL/Nd: YAG/lightsheer diode Workstation, Santa Clara, CA, USA AL: Cynosure, Westford, MA, USA	VSS	GOOD
										IPL: λ = 515–590, ED = 18–24; AL: λ = 755 with an aiming beam of λ = 543, S = 12, ED = 12.5			
Issler-Fisher [45]	2021	Case–control	Conventional management	5.1	187	75/112	39 (IQR 27–49)	UFCL	NA	ActiveFxTM (3–45% Density, 250–300 Hz, 80–125 mJ), DeepFxTM (5–15% Density, 300 Hz, 15–50 mJ) SCAAR FxTM mode (1–5% Density, 250 Hz, 60–150 mJ)	Ultrapulse, Lumenis	VSS, O-POSAS, P-POSAS, ST	GOOD
Joo [53]	2022	NRCT	UAC	7 days after the 3rd laser treatment	14	14/0	45.01 ± 15.03	Er:YAG (non-ablative fractional laser)	NA	λ = 1550, 70 mJ (at level 7, with eight passes)	Fraxel Restore; Solta Medical, Pleasanton, CA, USA	ST	FAIR
Kemp Bohan [58]	2021	IPCS	PC	2	21	17/4	30.0 (24.5–40.5)	UFCL	NA	15% density, 300 Hz, 15 or 17.5 mJ	Lumenis UltraPulse Ltd., Yokneam, Israel	ST	GOOD
Khandelwa [34]	2014	Cohort (retrospective)	PC	7	40	22/18	18 (1–70)	UFCL	NA	DeepFX™: 12.5–27.5 mJ, density of 15%. ActiveFX™: 90–125 mJ, density 3–5%.	Lumenis UltraPulse^®^, Santa Clara, CA	VSS	GOOD
Khedr [59]	2020	IPCS	PC	3	50	27/23	16.76 ± 7.72	Nd:YAG laser (*n* = 25) or E-light (IPL + radiofrequency) (*n* = 25)	NA	Nd: YAG: λ = 1064, ED = 45–75, PD = 25–45, S = 6. E-light:IPL: λ = 530–755, ED = 30–40, PD = 2–7 with 15–30 ms pulse delay, 8 mm × 32 mm hand piece. RF: 6–10 J/cm^3^	Nd: YAG laser (Cynergy; Cynosure Inc, Westford, MA), E-light (two-handle beauty machine; Beijing Oriental Wison Mechanical & Electronic Co., Ltd.)	VSS	GOOD
Kono [54]	2005	NRCT	UAC	1	15	8/7	13.7 (1–42)	Long-pulsed PDL	NA	Λ = 595, S = 7, ED = 9–10, PD = 1.5–10	model V-beam; Candela Laser Corporation, Wayland, MA	mVGH	FAIR
Lei [60]	2015	IPCS	PC	3.75	63	25/38	16–45	UFCL	Traditional Chinese medicine	P = 25~32 W, PD = 4~6, spacing 0.6~1.0 mm (for early stage of scar growth, 1.0 mm, decreasing with the number of treatments)	LJL35-CS Ultra Pulse CO_2_ Fractional Laser	VSS	GOOD
Li [35]	2021	Cohort (retrospective)	PC	1	165	79/86	3.5 ± 3.02	UCFL	NA	5% density, 30–50 mJ/microbeam exposure	Lumenis Ltd., Yokneam, Israel	VSS, ST	GOOD
Li * [43]	2021	Case–control (retrospective)	PC	12	105	46/59	39.5 ± 6.02	595 nm PDL	NA	PD = 0.45, ED = 5–9 (low ED: 5–7 and high ED = 7–9), S = 7	Vbeam Perfecta, Candela, USA	VSS, ST	GOOD
Li [36]	2018	Cohort (retrospective)	PC	1–2	221	81/140	3–48 (29 ± 8)	595-PDL or UFCL	NA	PDL: λ = 595, S = 7, PD = 0.45~1.50, ED = 5.0~7.0. CO_2_: For scar height <2 mm, choose Deep FX mode (5~10% density, 25~50 mJ); for scar height ≥2 mm, choose Scaar FX mode (3~5% density, 60~120 mJ)	PDL: Vbeam Perfecta, Candela, USA; CO_2_: Ultra Pulse CO_2_ Fractional Laser (Cornmedical Medical Laser, Inc., USA)	VSS, VAS	GOOD
Liu [44]	2022	Case–control (retrospective)	Conventional surgery	1–1.5	46	28/18	38.22 ± 10.28	UFCL	NA	3–5% density, 20–150 MJ, depth: 0.4–4 mm	UltraPulse^®^ Encore; Lumenis™	P-POSAS	GOOD
Majid [61]	2018	IPCS (open-label study)	PC	3	10	3/7	9.7 (5–12)	AFCL	Topical corticosteroids	8.4% density, 90–150 mJ at 30 W	eCO_2_ Laser; Lutronics Corp., Seoul, South Korea	VSS	GOOD
Matuszczak [62]	2021	IPCS	PC	1.25–1.5	25	16/9	6.40 ± 1.72	PDL followed by AFCL	NA	PDL: λ = 595, ED = 6.86 (5–10); AFCL: 75.12 mJ (54–80 mJ).	PDL: Syneron Candela VBeam Perfecta, Wayland, MA; AFCL: Smaxe	VSS, P-POSAS	GOOD
Miletta [63]	2021	IPCS	PC	6	22	15/7	28 ± 16.8	UFCL	NA	5–10% density, 30–50 mJ, depth 1.2–2.2 mm	Lumenis Ltd., Yokneam, Israel	P-POSAS, O-POSAS, ST	GOOD
Patel [37]	2019	Cohort	PC	After all laser treatment	49	26/23	4.86 ± 4.5	UFCL	NA	First pass: SCAAR FX (majority): for scar depth: 1–3 mm: 1.7% density, 250 Hz, 101.6 mJ. Second pass: DeepFX for scar depth less than 1 mm: 5.3% density, 266.2 Hz, 13.7 mJ and ActiveFX (minority)	Ultrapulse, Lumenis	O-POSAS, P-POSAS, total POSAS	GOOD
Poetschke [64]	2017	IPCS	PC	6	10	3/7	39.3 ± 15.3	UFCL	NA	First: ScaarFX, shape 2, size 10, pulse 1, density 1%, repeat delay 0.3 s, 250 Hz, 70–120 mJ; Second: ActiveFX: 9% density, 40 mJ, 350 Hz, pattern 1, size 2, repeat delay 0.1 s. Finally: ActiveFX: 2% density, 100 mJ, 125 Hz, pattern 1, size 6, repeat delay 0.1 s	Ultrapulse, Lumenis	VSS, O-POSAS, P-POSAS	GOOD
Tan [38]	2021	Cohort (retrospective)	PC	1	221	118/103	33.6 ± 11.8	AFCL	NA	Deep mode: 5–10% density, 15–30 MJ, depth of 550–800 μm. Superficial mode: 40% density, 70–150 MJ, depth of 50–150 μm	AcuPulse; Lumenis Ltd., Yokneam, Israel	VSS	GOOD
Tawfic [65]	2020	IPCS	PC	3	25	2/23	22.04 ± 9.92	AFCL OR FMR (fractional microneedle radiofrequency)	NA	FMR: power level of eight to nine (max 70 w), an exposure time of 800 ms, a depth of 2 mm (using non-insulated needles, 2 Hz frequency, and 2 passes and diameters of 0.3 mm per needle). AFCL: 18–20 W, 800–1000 µs dwell time, 500–600 μm spacing (13% density), microspot size 120 μm, two to three stacks	FMR:VIVACE™ combine microneedling with bipolar RF; AFCL: DEKA Smartxide DOT, Italy	VSS, POSAS	GOOD
Tawfic [47]	2022	RCT	PC	1	20	2/18	24.80 ± 9.87 (16–48)	AFCL	NA	20 W, 800–1000 ms dwell time, and 2–3 stacks for scar thickness (low-density, 900 mm, medium-density, 600 mm, spacing (12.6% density), high-density, 300 mm spacing (25.6% density))	The DEKA; Smart Xide DOT, Calenzano, Italy	O-POSAS, P-POSAS	GOOD
Travis [39]	2022	Cohort	PC	1–2 weeks	23	16/13	49.1 (IQR: 36.7–58.6)	AFCL	NA	Firstly, SCAARFx modality 1% density, 70 mJ	Ultrapulse, Lumenis, Yokneam, Israel	VSS, O-POSAS, P-POSAS, total POSAS	GOOD
Wang [66]	2017	IPCS	PC	6	95	40/55	22.9 (12–55)	FMR (fractional microplasma radiofrequency)	NA	Roller tip at 50–80 watts; 3–4 passes in different directions over each area with a high rolling speed of 5 cm/s and a delay of 5–10 s between passes	Pixel RF, Accent XL; Alma Lasers, Caesarea, Israel	O-POSAS, P-POSAS, total POSAS	GOOD
Weshahy [67]	2020	IPCS	PC	2	15	8/7	38.95 ± 8.85	AFCL	Combined with growth factors in group AFCL + GF	Smart stack, dot mode, power: 30 W, dwell time: 800 ms, spacing: 400 μm and smart stacking: 2, depth 200 µm, S = 15, 17% density. Readymade GFs after sessions for at least 6 h	SmartXide DOT^®^; DEKA, Florence, Italy	VSS, O-POSAS, P-POSAS, total POSAS	GOOD
Woods [40]	2022	Cohort	PC	18 months after injury	21	NA	NA	PDL (13) or Nd:YAG Q-switched KTP laser (8) or combined	NA	PDL: λ = 595, S = 7–10, PD = 0.5–10, ED = 7–12, KTP: λ = 532, S = 2–6, ED = 1–6	V-Beam 595 pulsed dye laser (Candela), Nd: YAG Q-switched KTP laser (Cynosure)	Total POSAS	GOOD
Xie [41]	2018	Cohort	PC	11	221	118/103	8 (IQR: 4, 31)	PDL + AFCL	NA	PDL:λ = 595, S = 7–12, PD = 1.5–3.0, ED = 8.0~9.5. AFCL: For scar height < 1 mm, choose Deep FX mode: 5~10% density, 25~50 mJ; for scar height ≥1 mm, choose Scaar FX mode: 3~5% density, 80~150 mJ	PDL, Vbeam Platinum, Candela, USA	Self-made scar rating score (refer to the VSS), ST	GOOD
											AFCL: UltraPulse Encor, Lumenis, USA		
Yang [68]	2022	IPCS	PC	1	169	92/77	3.28 (1–6)	UFCL	NA	1. Scar height ≤2 mm, ActiveFX: 125 mJ, 50 Hz; Deepn FX: 25–50 mJ, 5–10% density	UltraPulse Encor, Lumenis, Santa Clara, CA, USA	VSS	GOOD
										2. Scar height ≥2 mm, ActiveFX: 150 mJ, 150 Hz; Deepn FX: 60–120 mJ, 3–5% density			
Yang [46]	2021	RCT	PC	3	20	13/7	26 (3–67)	PDL	NA	λ = 595, S = 7, ED = 5–7 PD = 0.45–1.50	America, Candela Company	VSS	GOOD
Zuccaro [42]	2021	Cohort	PC	12	32	13/7	5.89	AFCL or AFCL + PDL	NA	PDL: λ = 595, ED = 5.50 (5.00–7.00); PD = 0.45 (0.45–1.50)	PDL (Vbeam Perfecta, Candela Corporation, Wayland, MA, USA)	VSS, P–POSAS, O–POSAS	GOOD
										AFCL: deep: 5% density; 70.00 mJ	AFCL (CO_2_RE, Candela Corporation, Wayland, MA, USA)		
										Fusion: core energy: 70.00 mJ, ring energy: 48.00–55.20 mJ, 20–25% density			
Zuccaro [69]	2018	IPCS	PC	Differed for each patient	71	NA	6.62	PDL or AFCL or AFCL + PDL	NA	PDL:ED = 6.45 (5–9).	PDL: Syneron Candela Vbeam Perfecta, Wayland, MA, USA AFCL:Syneron Candela CO_2_RE, Wayland, MA, USA	VSS	GOOD
										AFCL: core energy 70.86 mJ (53–78 mJ); settings: fusion and deep modes (most-used)			

Randomized controlled trial (RCT); non-randomized controlled trial (NRCT); in-patient controlled study (IPCS); untreated area control (UAC); pretreatment control (PC); Vancouver Scar Scale (VSS); Patient and Observer Scar Assessment Scale (POSAS); visual analogue scale (VAS); scar thickness by ultrasonography (ST); modified Vancouver General Hospital (mVGH); ultrapulse fractional CO_2_ laser (UFCL); ablative fractional CO_2_ laser (AFCL); pulsed dye laser (PDL); low-level laser treatment (LLLT); intense pulsed light (IPL); IPL + radiofrequency (E-light); fractional microneedle radiofrequency (FMR); wavelength (nm) (λ); pulse duration (PD, in ms); energy density (J/cm^2^)(ED); spot size (mm) (S); not available (NA).* In order to distinguish between the two Li’s articles published in 2021, we have marked this one with an asterisk [43].

## Data Availability

The original data are included in the article. Further information can be obtained from the corresponding author.

## Supplementary Materials

The following supporting information can be downloaded at: https://www.mdpi.com/article/10.3390/ebj4020013/s1, Figure S1: Forest plot showing the effects of photo-electric therapy for burn scars according to VSS, POSAS, thickness, and VAS for the group with scarring for over one year and the group with scarring between six months and one year; File S1: the PRISMA-2020-Checklist [96,97,98].
