# Supplementary material for: Laser, Intense Pulsed Light, and Radiofrequency for the Treatment of Burn Scarring: A Systematic Review and Meta-Analysis
Source: Eur Burn J. 2023 Mar 23;4(2):142–62. doi: 10.3390/ebj4020013 (PMC11571841; doi:10.3390/ebj4020013)
Supplement: Supplementary file 1 [file ebj-04-00013-s001.zip › Figure S1.pdf]

Figure S1

Study or Subgroup	Experimental Mean	Experimental SD	Experimental Total	Control Mean	Control SD	Control Total	Weight	Std. Mean Difference IV, Random, 95% CI
**1.6.1 Over 1 year**								
Douglas 2019	31.47	12.75	19	33.53	10.74	19	2.8%	-0.17 [-0.81, 0.47]
El-Hoshy 2017	5.18	1.18	20	7.76	2.07	20	2.8%	-1.50 [-2.21, -0.79]
Elrod 2020	4.88	1.73	17	7.65	2.12	17	2.7%	-1.40 [-2.16, -0.64]
El-Zawahry 2015 HPTP	9	5.6	8	9	3	8	2.5%	0.00 [-0.98, 0.98]
El-Zawahry 2015 Keloid	17.5	11.4	3	13.5	2.1	3	1.9%	0.39 [-1.25, 2.03]
Gaida > 12m 2004	-0.344	1.42	5	-0.149	1.41	5	2.3%	-0.12 [-1.37, 1.12]
Hultman 2013	5.16	1.92	147	10.43	2.37	147	3.0%	-2.44 [-2.74, -2.13]
Issler-Fisher 2021	-0.919	2.1	167	-0.294	1.88	20	2.9%	-0.30 [-0.76, 0.17]
Khandelwal 2014	5.4	2.1	40	7.6	12.1	40	2.9%	-0.25 [-0.69, 0.19]
Kono 2005	3.6	1.34	15	6.8	0.84	15	2.5%	-2.78 [-3.83, -1.74]
Li > 12m 2021*	4.31	1.44	11	8.82	1.17	11	2.2%	-3.31 [-4.67, -1.94]
Majid 2018	2	2.36	10	7	1.6	10	2.3%	-2.38 [-3.58, -1.17]
Miletta 2021	0.95	0.33	22	1.35	0.48	22	2.8%	-0.95 [-1.58, -0.33]
Patel 2019	69.19	14.9	49	89.57	17.5	49	2.9%	-1.24 [-1.68, -0.81]
Poetschke 2017	13.3	2.869	10	23.6	10.9	10	2.5%	-1.24 [-2.21, -0.26]
Tan > 12m 2021	7.7	2	55	9.2	1.8	55	3.0%	-0.78 [-1.17, -0.39]
Tawfic AFCL 2022	14.25	2.69	20	20.1	5.24	20	2.8%	-1.38 [-2.07, -0.68]
Tawfic FMR 2022	14.85	3.5	20	22.3	5.53	20	2.8%	-1.58 [-2.30, -0.86]
Tawfic high density 2020	2.56	0.82	25	7.64	1.35	25	2.5%	-4.48 [-5.55, -3.41]
Tawfic low density 2020	3.96	1.43	25	7.64	1.35	25	2.7%	-2.60 [-3.37, -1.84]
Tawfic mediun density 2020	3.16	1.28	25	7.64	1.35	25	2.6%	-3.35 [-4.23, -2.47]
Travis 2022	7.42	2.57	23	8.58	1.55	23	2.9%	-0.54 [-1.13, 0.05]
Wang 2017	46.35	5.3	95	53.41	6.28	95	3.0%	-1.21 [-1.52, -0.90]
Weshahy AFCL + GF 2020	17.95	2.73	15	36	4.69	15	2.1%	-4.58 [-6.01, -3.14]
Weshahy AFCL only 2020	21.1	2.91	15	36.95	4.69	15	2.3%	-3.95 [-5.24, -2.66]
Woods 2022	38.14	17.33	21	54.28	12.84	21	2.8%	-1.04 [-1.69, -0.39]
Zuccaro > 12m 2018	5	2	34	6.11	2.4	34	2.9%	-0.50 [-0.98, -0.01]
**Subtotal (95% CI)**			**916**			**769**	**71.6%**	**-1.55 [-1.94, -1.15]**
Heterogeneity: Tau² = 0.90; Chi² = 268.60, df = 26 (P < 0.00001); I² = 90%								
Test for overall effect: Z = 7.70 (P < 0.00001)								
**1.6.2 6m-1y**								
Gaida < 12m 2004	-1.65	2.1	14	-0.094	1.82	14	2.7%	-0.77 [-1.54, 0.00]
Huang ACFL only 2021	7.8	0.6	12	13.8	0.6	12	1.0%	-9.66 [-12.75, -6.56]
Huang AFCL + AFI 2021	4.5	0.4	12	13.5	0.7	12	0.5%	-15.24 [-20.03, -10.46]
Joo 2022	0	0.41	88	0.05	0.99	88	3.0%	-0.07 [-0.36, 0.23]
Kemp Bohan 2021	0.41	0.3	21	0.71	0.43	21	2.8%	-0.79 [-1.42, -0.16]
Khedr Elight 2020	2.08	0.86	25	9.12	1.33	25	2.2%	-6.19 [-7.57, -4.81]
Khedr Nd 2020	3.86	1.09	25	8.88	2.14	25	2.7%	-2.91 [-3.72, -2.10]
Li > 6m 2021	7.17	2.02	43	9.25	1.81	43	2.9%	-1.07 [-1.53, -0.62]
Matuszczak 2021	14.26	5.98	25	28.83	9.21	25	2.8%	-1.85 [-2.52, -1.18]
Tan 6 − 12m 2021	7.9	1.7	34	8.8	1.9	34	2.9%	-0.49 [-0.98, -0.01]
Zuccaro 2021	4.85	0.53	20	8.62	0.54	20	1.9%	-6.91 [-8.62, -5.20]
Zuccaro < 12m 2018	6.52	2.34	36	8.63	1.79	36	2.9%	-1.00 [-1.49, -0.51]
**Subtotal (95% CI)**			**355**			**355**	**28.4%**	**-2.72 [-3.62, -1.81]**
Heterogeneity: Tau² = 2.11; Chi² = 234.43, df = 11 (P < 0.00001); I² = 95%								
Test for overall effect: Z = 5.89 (P < 0.00001)								
**Total (95% CI)**			**1271**			**1124**	**100.0%**	**-1.81 [-2.18, -1.43]**
Heterogeneity: Tau² = 1.20; Chi² = 515.49, df = 38 (P < 0.00001); I² = 93%								
Test for overall effect: Z = 9.41 (P < 0.00001)								
Test for subgroup differences: Chi² = 5.43, df = 1 (P = 0.02), I² = 81.6%								

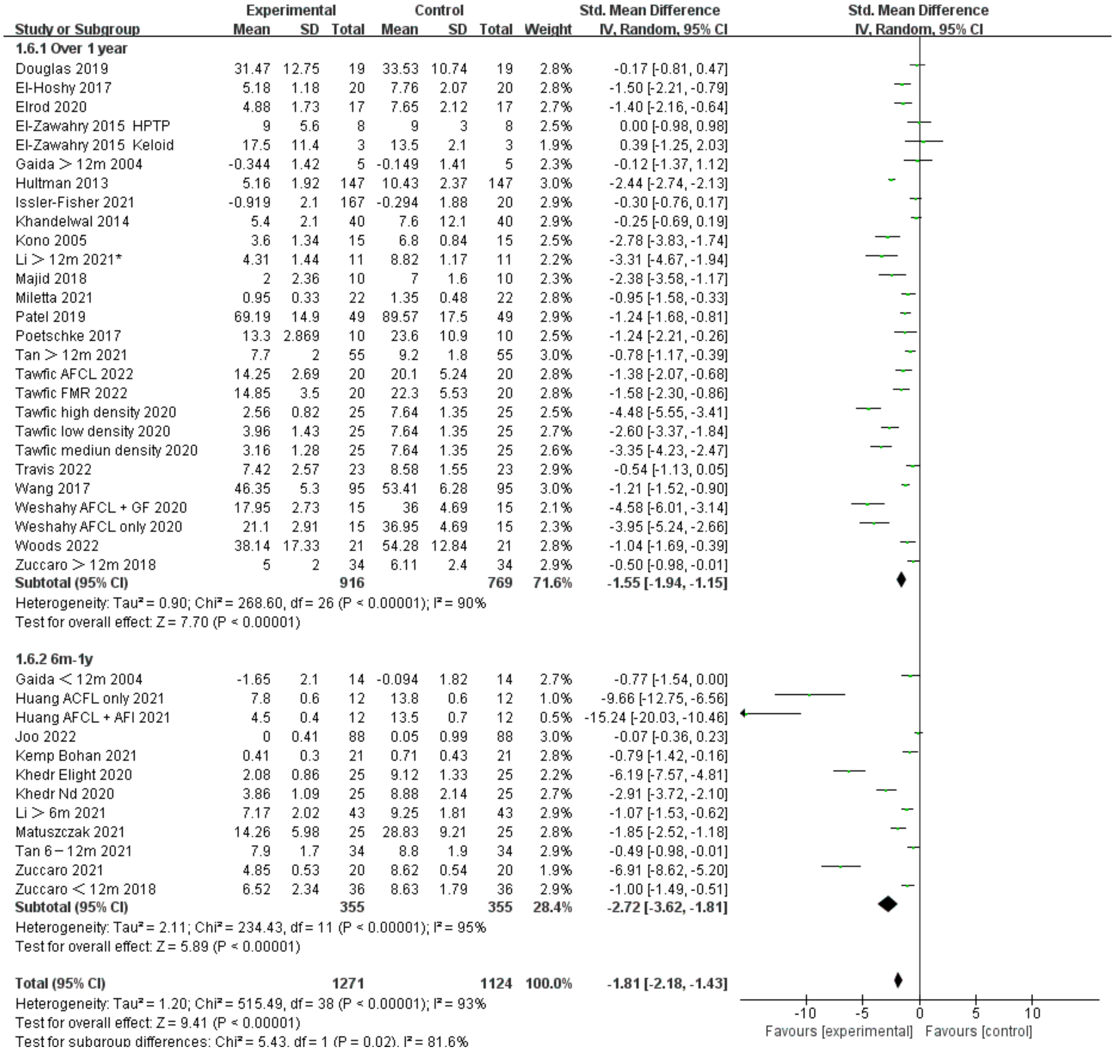

Figure S1. Forest plot showing the effects of photo-electric therapy for burn scars with different scar durations according to VSS, POSAS, thickness, and VAS for the group with scar.
