# Supplementary material for: Laser, Intense Pulsed Light, and Radiofrequency for the Treatment of Burn Scarring: A Systematic Review and Meta-Analysis
Source: Eur Burn J. 2023 Mar 23;4(2):142–62. doi: 10.3390/ebj4020013 (PMC11571841; doi:10.3390/ebj4020013)
Supplement: Supplementary file 1 [file ebj-04-00013-s001.zip › PRISMA_2020_checklist .pdf]

PRISMA 2020 Checklist

Section and Topic	Item #	Checklist item	Location where item is reported
**TITLE**			
Title	1	Identify the report as a systematic review.	Title
**ABSTRACT**			
Abstract	2	See the PRISMA 2020 for Abstracts checklist.	
**INTRODUCTION**			
Rationale	3	Describe the rationale for the review in the context of existing knowledge.	1 Introduction P(paragraph) 2
Objectives	4	Provide an explicit statement of the objective(s) or question(s) the review addresses.	Introduction P2
**METHODS**			
Eligibility criteria	5	Specify the inclusion and exclusion criteria for the review and how studies were grouped for the syntheses.	In 2.2 selection inclusion
Information sources	6	Specify all databases, registers, websites, organisations, reference lists and other sources searched or consulted to identify studies. Specify the date when each source was last searched or consulted.	In 2.1 search strategy
Search strategy	7	Present the full search strategies for all databases, registers and websites, including any filters and limits used.	Table1 and the picture below
Selection process	8	Specify the methods used to decide whether a study met the inclusion criteria of the review, including how many reviewers screened each record and each report retrieved, whether they worked independently, and if applicable, details of automation tools used in the process.	Two review authors independently selected studies. A third review author arbitrated if there were disagreements.
Data collection process	9	Specify the methods used to collect data from reports, including how many reviewers collected data from each report, whether they worked independently, any processes for obtaining or confirming data from study investigators, and if applicable, details of automation tools used in the process.	In 2.3 Data extraction
Data items	10a	List and define all outcomes for which data were sought. Specify whether all results that were compatible with each outcome domain in each study were sought (e.g. for all measures, time points, analyses), and if not, the methods used to decide which results to collect.	In 2.3 Data extraction
	10b	List and define all other variables for which data were sought (e.g. participant and intervention characteristics, funding sources). Describe any assumptions made about any missing or unclear information.	In 2.3 Data extraction
Study risk of bias assessment	11	Specify the methods used to assess risk of bias in the included studies, including details of the tool(s) used, how many reviewers assessed each study and whether they worked independently, and if applicable, details of automation tools used in the process.	In 2.4Methodological quality assessment of included studies
Effect measures	12	Specify for each outcome the effect measure(s) (e.g. risk ratio, mean difference) used in the synthesis or presentation of results.	In 2.5 Statistical analysis
Synthesis methods	13a	Describe the processes used to decide which studies were eligible for each synthesis (e.g. tabulating the study intervention characteristics and comparing against the planned groups for each synthesis (item #5)).	tabulating the study intervention characteristics and comparing against the planned groups for each synthesis.

Section and Topic	Item #	Checklist item	Location where item is reported
	13b	Describe any methods required to prepare the data for presentation or synthesis, such as handling of missing summary statistics, or data conversions.	In 2.5 Statistical analysis and using the items from Cochrane to do the data conversions.
	13c	Describe any methods used to tabulate or visually display results of individual studies and syntheses.	Forest plot IN Review Manager 5.4
	13d	Describe any methods used to synthesize results and provide a rationale for the choice(s). If meta-analysis was performed, describe the model(s), method(s) to identify the presence and extent of statistical heterogeneity, and software package(s) used.	In 2.5 Statistical analysis.<br>Std. mean difference (SMD) was used for the merging of multiple types of data. MD was used for combing the results of scar thickness. We used random effects for all.
	13e	Describe any methods used to explore possible causes of heterogeneity among study results (e.g. subgroup analysis, meta-regression).	Subgroup analysis
	13f	Describe any sensitivity analyses conducted to assess robustness of the synthesized results.	We changed to fixed effect to assess robustness
Reporting bias assessment	14	Describe any methods used to assess risk of bias due to missing results in a synthesis (arising from reporting biases).	In 3.4 Publication bias
Certainty assessment	15	Describe any methods used to assess certainty (or confidence) in the body of evidence for an outcome.	Oxford Centre for Evidence-Based Medicine (OCEBM) assessment[1]
**RESULTS**			
Study selection	16a	Describe the results of the search and selection process, from the number of records identified in the search to the number of studies included in the review, ideally using a flow diagram.	Figure 1
	16b	Cite studies that might appear to meet the inclusion criteria, but which were excluded, and explain why they were excluded.	Figure 1 [2],unable to obtain complete data.
Study characteristics	17	Cite each included study and present its characteristics.	Table 2
Risk of bias in studies	18	Present assessments of risk of bias for each included study.	Table 2
Results of individual studies	19	For all outcomes, present, for each study: (a) summary statistics for each group (where appropriate) and (b) an effect estimate and its precision (e.g. confidence/credible interval), ideally using structured tables or plots.	Figure.2-6

PRISMA 2020 Checklist

Section and Topic	Item #	Checklist item	Location where item is reported
Results of syntheses	20a	For each synthesis, briefly summarise the characteristics and risk of bias among contributing studies.	Table.2
	20b	Present results of all statistical syntheses conducted. If meta-analysis was done, present for each the summary estimate and its precision (e.g. confidence/credible interval) and measures of statistical heterogeneity. If comparing groups, describe the direction of the effect.	Figure.2-6
	20c	Present results of all investigations of possible causes of heterogeneity among study results.	Subgroup analysis in Figure.2-6
	20d	Present results of all sensitivity analyses conducted to assess the robustness of the synthesized results.	Changed to fixed analysis to assess the robustness. The results are positive same as random effect.
Reporting biases	21	Present assessments of risk of bias due to missing results (arising from reporting biases) for each synthesis assessed.	In 3.4 Publication bias
Certainty of evidence	22	Present assessments of certainty (or confidence) in the body of evidence for each outcome assessed.	LEVEL 3 of OCEBM[1]
**DISCUSSION**			
Discussion	23a	Provide a general interpretation of the results in the context of other evidence.	4 Discussion part P1-2
	23b	Discuss any limitations of the evidence included in the review.	6 Limitations
	23c	Discuss any limitations of the review processes used.	Only studies with positive findings published and the heterogeneity in parameters and different treatments and different assessments.
	23d	Discuss implications of the results for practice, policy, and future research.	5 Conclusion
**OTHER INFORMATION**			
Registration and protocol	24a	Provide registration information for the review, including register name and registration number, or state that the review was not registered.	CRD42023397244
	24b	Indicate where the review protocol can be accessed, or state that a protocol was not prepared.	PROSPERO
	24c	Describe and explain any amendments to information provided at registration or in the protocol.	None
Support	25	Describe sources of financial or non-financial support for the review, and the role of the funders or sponsors in the review.	This research received no external funding
Competing interests	26	Declare any competing interests of review authors.	The authors state no conflict of interest.
Availability of data, code and other materials	27	Report which of the following are publicly available and where they can be found: template data collection forms; data extracted from included studies; data used for all analyses; analytic code; any other materials used in the review.	Data extracted from included studies

From: Page MJ, McKenzie JE, Bossuyt PM, Boutron I, Hoffmann TC, Mulrow CD, et al. The PRISMA 2020 statement: an updated guideline for reporting systematic reviews. BMJ 2021;372:n71. doi: 10.1136/bmj.n71
For more information, visit: http://www.prisma-statement.org/

[1] OCEBM Levels of Evidence Work Group. (n.d.). The Oxford Levels of Evidence 2. Oxford Center of Evidence Based Medicine. https://www.cebm.ox.ac.uk/resources/levels-of-evidence/ ocebm-levels-of-evidence.
[2] L.E. Cooper, K. Nuutila, P.M. Kemp Bohan, V. Diaz, M. Batchinsky, A.H. Carlsson, L.C. Cancio, R.K. Chan, Analysis of the Utility of CO2 and Pulse-Dye Lasers Together and Separately in the Treatment of Hypertrophic Burn Scars, Ann Plast Surg, 89 (2022) 166-172.

Search strategy

PUBMED

- ("**laser*"[**Title/Abstract] OR "fractional carbon dioxide"[Title/Abstract] OR "fractional co2"[Title/Abstract] OR "pulsed dye laser"[Title/Abstract] OR "PDL"[Title/Abstract] OR "Biostimulation"[Title/Abstract] OR "Photobiomodulation"[Title/Abstract] OR "low level laser"[Title/Abstract] OR "LLLT"[Title/Abstract] OR "Neodymium Doped Yttrium Aluminum Garnet"[Title/Abstract] OR "er yag"[Title/Abstract] OR " erbium "[Title/Abstract] OR "nd yag"[Title/Abstract] OR "**light**"[Title/Abstract] OR "phototherap*"[Title/Abstract] OR "intense pulsed light"[Title/Abstract] OR "IPL"[Title/Abstract] OR "**radiofrequency**"[Title/Abstract] OR "radio-frequency"[Title/Abstract]) AND ("**therap*"[**Title/Abstract] OR "treatment*"[Title/Abstract] OR "therapeutic*"[Title/Abstract]) AND ("**burn*"[**Title/Abstract] OR "postburn"[Title/Abstract] OR "post burn"[Title/Abstract]) AND ("**Cicatrix**"[Title/Abstract] OR "Scar"[Title/Abstract] OR "Scars"[Title/Abstract] OR "Cicatrization"[Title/Abstract] OR "Scarring"[Title/Abstract] OR "hypertrophic scar*"[Title/Abstract] OR "keloid"[Title/Abstract])
- Filters: Clinical Study, Humans

EMBASE

- **('phototherapy'/exp OR ((**'fractional carbon dioxide':ti,ab,kw OR 'fractional co2':ti,ab,kw OR 'pulsed dye laser':ti,ab,kw OR 'pdl':ti,ab,kw OR 'biostimulation':ti,ab,kw OR 'photobiomodulation':ti,ab,kw OR 'low level laser':ti,ab,kw OR 'lllt':ti,ab,kw OR 'neodymium doped yttrium aluminum garnet':ti,ab,kw OR 'er yag':ti,ab,kw OR 'erbium':ti,ab,kw OR 'nd yag':ti,ab,kw OR 'intense pulsed light':ti,ab,kw OR 'ipl':ti,ab,kw OR 'radiofrequency'/exp OR 'radio-frequency':ti,ab,kw) AND (**'therapy'/exp** OR 'therap*':ti,ab,kw OR 'treatment*':ti,ab,kw OR 'therapeutic*':ti,ab,kw))) AND (**'burn scar'/exp** OR (('burn*':ti,ab,kw OR 'postburn':ti,ab,kw OR 'post-burn':ti,ab,kw) AND ('cicatrix':ti,ab,kw OR 'scar':ti,ab,kw OR 'scars':ti,ab,kw OR 'cicatrization':ti,ab,kw OR 'scarring':ti,ab,kw OR 'hypertrophic scar*':ti,ab,kw OR 'keloid':ti,ab,kw)))
- AND [humans]/lim AND [clinical study]/lim

cochrane

- #1 MeSH descriptor: [Cicatrix] explode all trees
- #2 MeSH descriptor: [Phototherapy] explode all trees
- #3 MeSH descriptor: [Radiofrequency Therapy] explode all trees
- #4 MeSH descriptor: [Burns] explode all trees
- #5 (cicatrix OR Scar OR Scars OR Cicatrization OR Scarring OR (hypertrophic scar) OR keloid):ti,ab,kw
- #6 (burn* OR postburn OR (post-burn)):ti,ab,kw
- #7 (('fractional carbon dioxide' OR 'fractional co2' OR 'pulsed dye laser' OR 'pdl' OR 'biostimulation' OR 'photobiomodulation' OR 'low level laser' OR 'lllt' OR 'neodymium doped yttrium aluminum garnet' OR 'er yag' OR 'erbium' OR 'nd yag' OR 'intense pulsed light' OR 'ipl' OR 'radio-frequency') AND ('therapy' OR 'therap*' OR 'treatment*' OR 'therapeutic*')):ti,ab,kw
- #8 (#4 OR #6) AND (#1 OR #5)
- #9 #2 OR #3 OR #7
- #10 #8 AND #9
